# *Brucella abortus* Uses a Stealthy Strategy to Avoid Activation of the Innate Immune System during the Onset of Infection

**DOI:** 10.1371/journal.pone.0000631

**Published:** 2007-07-18

**Authors:** Elías Barquero-Calvo, Esteban Chaves-Olarte, David S. Weiss, Caterina Guzmán-Verri, Carlos Chacón-Díaz, Alexandra Rucavado, Ignacio Moriyón, Edgardo Moreno

**Affiliations:** 1 Programa de Investigación en Enfermedades Tropicales, Escuela de Medicina Veterinaria, Universidad Nacional, Heredia, Costa Rica; 2 Centro de Investigación en Enfermedades Tropicales, Facultad de Microbiología, Universidad de Costa Rica, San José, Costa Rica; 3 Department of Microbiology and Skirball Institute, New York University School of Medicine, New York, New York, United States of America; 4 Instituto Clodomiro Picado, Facultad de Microbiología, Universidad de Costa Rica, San José, Costa Rica; 5 Department of Microbiology, University of Navarra, Navarra, Spain; University of California at Merced, United States of America

## Abstract

**Background:**

To unravel the strategy by which *Brucella abortus* establishes chronic infections, we explored its early interaction with innate immunity.

**Methodology/Principal Findings:**

*Brucella* did not induce proinflammatory responses as demonstrated by the absence of leukocyte recruitment, humoral or cellular blood changes in mice. *Brucella* hampered neutrophil (PMN) function and PMN depletion did not influence the course of infection. *Brucella* barely induced proinflammatory cytokines and consumed complement, and was strongly resistant to bactericidal peptides, PMN extracts and serum. *Brucella* LPS (*Br*LPS), NH-polysaccharides, cyclic glucans, outer membrane fragments or disrupted bacterial cells displayed low biological activity in mice and cells. The lack of proinflammatory responses was not due to conspicuous inhibitory mechanisms mediated by the invading *Brucella* or its products. When activated 24 h post-infection macrophages did not kill *Brucella*, indicating that the replication niche was not fusiogenic with lysosomes. *Brucella* intracellular replication did not interrupt the cell cycle or caused cytotoxicity in WT, TLR4 and TLR2 knockout cells. TNF-α-induction was TLR4- and TLR2-dependent for live but not for killed *B. abortus*. However, intracellular replication in TLR4, TLR2 and TLR4/2 knockout cells was not altered and the infection course and anti-*Brucella* immunity development upon *Br*LPS injection was unaffected in TLR4 mutant mice.

**Conclusion/Significance:**

We propose that *Brucella* has developed a stealth strategy through PAMPs reduction, modification and hiding, ensuring by this manner low stimulatory activity and toxicity for cells. This strategy allows *Brucella* to reach its replication niche before activation of antimicrobial mechanisms by adaptive immunity. This model is consistent with clinical profiles observed in humans and natural hosts at the onset of infection and could be valid for those intracellular pathogens phylogenetically related to *Brucella* that also cause long lasting infections.

## Introduction

Pathogenic bacteria use a variety of virulence factors that endow them with the ability to overcome the immune system. Adhesins, enzymes and toxins acting on host tissues, cells and free molecules enable pathogens to breach host barriers and thwart defenses. In most cases, however, the aggression is quickly sensed by innate immune defenses that both act immediately and bolster the adaptive immune response. Innate immunity detects minute amounts of components bearing the so-called pathogen-associated molecular patterns (PAMPs) as well as some products of host damage [1]. The subsequent proinflammatory responses are manifestations of the innate immunity and usual landmarks of infection and septic syndromes. However, there is increasing evidence that some pathogens display altered PAMPs in key molecules, suggesting that to escape detection by innate immunity is a survival strategy. One of the best examples of a structure with altered PAMPs is the lipopolysaccharide (LPS) of *Brucella* (*Br*LPS), an intracellular parasite of worldwide importance [2]. *Br*LPS bears a non-canonical lipid A and, although it signals through toll-like receptor (TLR) 4 [3], it is active only at very high concentrations [4], [5]. Moreover, *Br*LPS confers a highly resistant phenotype to cationic bactericidal peptides and makes *Brucella* a poor activator of the complement system [4], [6]. Accordingly, we have suggested that evading innate immunity is decisive for *Brucella* parasitism [5], [7]. Yet, there are conflicting reports on the role of TLR4 and TLR2 in the immunity against live *Brucella* [8]–[12]. Although the course of experimental brucellosis seems unaltered in TLR2 knockout (KO) mice [8], [11], [12], the receptor was shown to be involved in the detection of heat-killed (HK) *Brucella abortus* [13]. Furthermore, some authors reported that *Brucella* lipoproteins (BLPs) are potent triggers of proinflammatory cytokines through TLR2, and proposed that *B. abortus* stimulates the innate immune system and induces cytokine-mediated inflammation by this mechanism [14]. Similarly, other authors have reported that *Brucella* replicates to a higher extend in TLR4 KO mice than in the WT [8], [12], while others do not notice significant differences of *Brucella* replication in these mice [9]–[11].

These discrepancies are not trivial. At the onset of the infection, brucellosis courses without significant endotoxicity signs or significant blood changes [15], an unusual fact that calls for an experimentally supported explanation. Moreover, *Brucella* lacks classical virulence factors [16], although it possesses a type IV secretion system VirB and periplasmic cyclic β-1,2-glucans that enable the pathogen to reach its final replicating niche in the endoplasmic reticulum [17], [18]. In addition, BvrR/BvrS, a two component sensory and regulatory system essential for *Brucella* virulence, controls the outer membrane (OM) composition and possibly aspects of the pathogen metabolism [19], [20]. Since activated macrophages successfully deal with intracellular *Brucella* [21], [22] it may be that prevention of early host cell activation is a prerequisite for β-glucans, VirB and other factors to become effective. To test this hypothesis, we have investigated the proinflammatory responses induced by *B. abortus* in the murine model in comparison with *Salmonella typhimurium*. We show here that *B. abortus* behaves in fact as a furtive pathogen that circumvents proinflammatory responses and propose that this strategy does not rely on inhibitory mechanisms but rather on the negligible activity of those molecules that bear marked PAMPs in other bacteria. The data presented are consistent with clinical profiles at the onset of brucellosis and argue that this model could be valid for those intracellular pathogens phylogenetically related to *Brucella* that also cause long lasting infections.

## Results

### *B. abortus* infected mice do not show symptoms of sepsis

As reported before [23], [24], *S. typhimurium* induced symptoms of septic shock in mice that started 2 h after intraperitoneal injection of 10^5^ CFU and were initially characterized by piloerection, decrease in feeding and water consumption and general malaise. At later times, signs progressed to severe wasting and cachectia, with death before 5 days. Pathological and histopathological examination of the lungs demonstrated alveolar edema, hemorrhage, extensive air space damage and PMN infiltration (not shown). None of these symptoms were observed in mice injected intraperitoneally with live- or HK-*B. abortus* in doses ranging from 10^4^ to 10^7^ CFU doses. However, bacteria were isolated from blood as early as 1 h of infection and bacteremia persisted for at least 48 h. Spleen cellular profiles and weights between *B. abortus*-infected with 10^5^ to 10^7^ colony forming units (CFU) and PBS-injected mice did not differ significantly in the first 48 h post-inoculation. Peritoneum and spleen of *B. abortus* infected mice (10^4^–10^5^ CFU) had close to one log increase in CFU at 24h of infection. Infection doses of 5×10^9^ to 10^10^ CFU of *B. abortus* did not induce the classical endotoxic shock profile observed with *Salmonella*, although these bacterial quantities were lethal for 50–60% of the mice after 48 h.

### *B. abortus* infected mice do not demonstrate acute coagulopathy disorders

**Figure 1:**
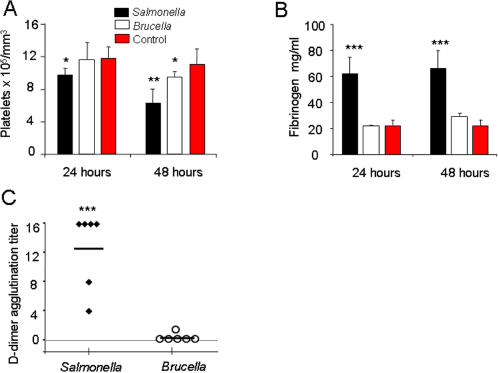
*B. abortus* does not induce augmented levels of fibrinogen, fibrin-breakdown products or important platelet aggregation. Balb/c mice (6 mice per group) were intraperitoneally injected with 10^6^ CFU of *B. abortus* 2308, 10^5^ CFU *S. typhimurium* (6 mice per group) or 0.1 ml of PBS (10 mice per group) and blood was collected from the retro-orbital sinus and the blood from the various individuals subjected to analysis. (A) The number of platelets was determined by flow cytometry. (B) The levels of fibrinogen were determined in plasma. (C) The levels of fibrin D-dimers in the plasma from infected and PBS injected control mice were determined by agglutination of sensitized beads, after 48 h pos-infection. Minimum positive cut-off (0.5 µg/ml) is represented with a dashed line. Values of p<0.05 (*), p<0.005 (**) and p<0.0005 (***) are indicated.

Two critical events during strong proinflammatory responses are platelet aggregation and synthesis of acute response proteins such as fibrinogen [25]. Plasmin generation and the subsequent degradation of fibrin are also linked to proinflammatory responses and PMN activation [26]. Whereas *S. typhimurium* induced statistically significant thrombocytopenia already 24 h of infection, for *B. abortus* this was observed only after 48 h and at a less markedly level (Figure 1 A). Likewise, *S. typhimurium-* but not *B. abortus*-infected mice showed increased fibrinogen synthesis (Figure 1 B) and generation of high levels of plasmin activity (Figure 1 C).

### *B. abortus* infection does not induce leukocytosis or recruitment of PMN

**Figure 2:**
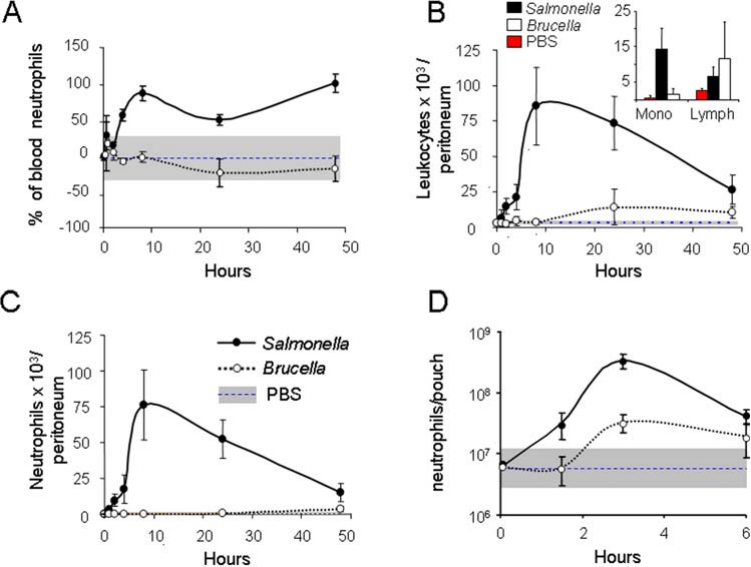
*B. abortus* does not induce leukocytosis or significant recruitment of PMN. Leukocyte counts were determined in the peritoneal fluids, heparinized blood or in the air pouch of Balb/c mice intraperitoneally injected with 10^6^ CFU *B. abortus* 2308, 10^5^ CFU *S. typhimurium* or 0.1 ml PBS. (A) Blood PMN were counted in 8 mice/group, during different periods. (B) Leukocyte values in the peritoneum were determined from fluids of 5 mice/group, in time. The inserted graph indicates the values of peritoneal lymphocytes and monocytes at 24 h. (C) The peritoneal PMN recruited were determined as in “(B)”. (D) PMN in air pouches were determined from the fluids of 5 mice/group, during 4 periods. PMN average numbers of PBS injected mice in each period (blue-dashed line) and the ranges of normal maximum upper and lower limits are depicted in each graphic (gray bar).

Gram negative bacterial infections commonly generate leukocytosis and PMN recruitment at early times. Thus, we explored the leukocyte blood changes and PMN recruitment in mice infected with *B. abortus* or *S. typhimurium*. As expected, the latter bacteria induced noticeable blood neutrophilia (Figure 2 A) and a significant recruitment of PMN and monocytes in the peritoneum (Figure 2 B and C) and in the air pouch model in mice (Figure 2 D). In some cases, *S. typhimurium* also caused neutrophilia at early stages followed by severe blood leucopenia starting at 10 h post-inoculation (not shown). *B. abortus* did not induce significant leukocyte blood changes or recruitment of PMN, monocytes or lymphocytes in the peritoneum of mice, and only a mild recruitment of PMN was observed in air pouches.

### PMN do not play a significant role in the clearance of *B. abortus in vivo*

**Figure 3:**
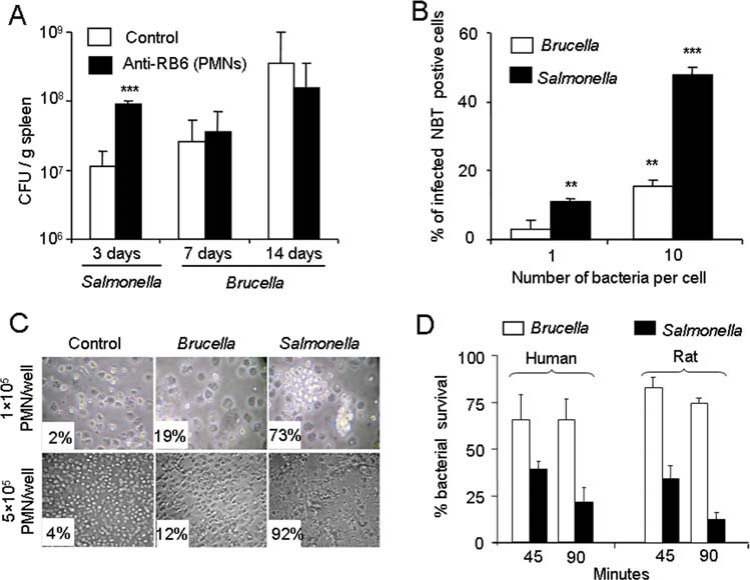
PMN are not required for innate control of *B. abortus* infection. (A) Balb/c mice were given repeated injections of the anti-RB6 antibody to deplete PMN. Untreated or PBS injected mice were used as controls. Two days later, all mice were intraperitoneally infected with 10^6^ CFU of *B. abortus* 2308 or 10^5^ *S. typhimurium*. During the infection, mice were treated with anti-RB6 antibody every two days, for a maximum of 7 days. Spleen bacterial counts were determined 3 days of infection for *Salmonella* and 7 and 14 days of infection for *B. abortus*. (B) *In situ* respiratory burst was estimated as the rate of *Brucella* or *Salmonella* infected rat PMN containing NBT positive granules at proportion of 1 or 10 bacteria/cell. (C) Degranulation of infected rat PMN (5±4 bacteria/cell) at two cell densities/well was estimated by microscopic examination (40× upper panel and 20× lower panel) at 3 h and expressed as the proportion of degranulated cells versus intact PMN in 5 fields. Standard error was less than 10% in all cases. (D) The rate of *B. abortus* or *S. typhimurium* survival in human and rat PMN was tested at 5±4 bacteria/cell, at two post infection times. Values of p<0.005 (**) and p<0.0005 (***) are indicated.

The role of PMN during *Brucella* infections has not been critically examined. We studied this aspect of innate immunity using mice chronically depleted of PMN by treatment with monoclonal antibody RB6. Bacterial counts were carried out 3 days of infection for *S. typhimurium* and 7 and 14 days for *B. abortus*, times at which these bacteria attain significant numbers in the spleen [11]. Consistent with previous reports, *S. typhimurium* replicated to a larger extent in PMN depleted than in control mice [27]. In contrast, *B. abortus* spleen CFU were similar in both groups after 7 or 14 days of infection (Figure 3 A). Since *Brucella* colonization of spleen and other organs takes place during the first hours [28], [29] and the phagocytosis of PMN starts immediately after bacterial invasion [30], these results show that the role of PMN in the control of *B. abortus* is not significant, even once adaptive immunity has developed.

### PMN are not significantly active against *B. abortus ex vivo*

It has been shown that virulent smooth brucellae hamper PMN degranulation and are more resistant than LPS defective strains to the killing action of PMN [31]. These properties, however, have not been weighed against those of other facultative intracellular gram negative bacteria. As expected [32], *S. typhimurium* stimulated the respiratory burst (Figure 3 B), did not prevent PMN degranulation (Figure 3 C) and was readily killed by these rat cells (Figure 3 D). Under the same conditions, *B. abortus* induced only a mild respiratory burst and a modest PMN degranulation (Figure 3 B and C), two facts more evident at rates of infection of 8–10 *B. abortus* per PMN than at lower rates. The inhibitory effect on degranulation lasted for up to 6 h post inoculation, a time when all control and *S. typhimurium* infected rat PMN were degranulated. Consistent with these observations, *B. abortus* was resistant to the killing action of human and rat PMN (Figure 3 D).

### *B. abortus* barely consumes complement and is resistant to bactericidal molecules

**Figure 4:**
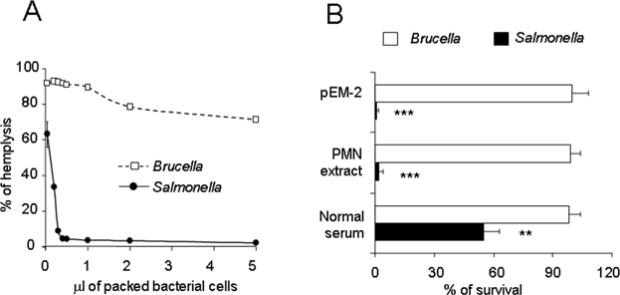
*B. abortus* does not consume complement and is resistant to PMN extracts, cationic peptides and serum. (A) Packed bacteria were incubated with normal rabbit serum and the remaining hemolytic activity of complement in serum measured in a complement fixation indicator system: higher hemolytic activity corresponds to less complement consumption by the bacteria. (B) Bactericidal activity was determined by incubating 4 × 10^5^ CFU of bacteria with 5 µM of cationic peptide pEM-2 or 5 mg/ml of PMN-extract in 0.2 ml PBS-1 % peptone, for 20 and 30 min. Complement bactericidal activity was estimated on 10^5^ CFU/ml bacterial suspensions dispensed in wells of microtiter plates (45 µl/well) containing fresh normal human serum (45 µl/well). Bactericidal action was estimated as the percentage of CFU with respect to controls without pEM-2, PMN extract or decomplemented inactivated serum, respectively. Values of p<0.005 (**) and p<0.0005 (***) are indicated.

Since complement activation and intracellular killing by microbicidal molecules are events linked to proinflammatory mechanisms, we compared the ability of *B. abortus* and *S. typhimurium* to consume complement and their resistance to cationic peptides, PMN extracts and normal serum. In contrast to *S. typhimurium*, *B. abortus* barely displayed anticomplementary activity (Figure 4 A) and it was considerable more resistant to the action of bactericidal cationic peptide p-EM2, PMN-extracts and normal serum (Figure 4 B).

### *B. abortus* infection induces minimal levels of cytokines

**Figure 5:**
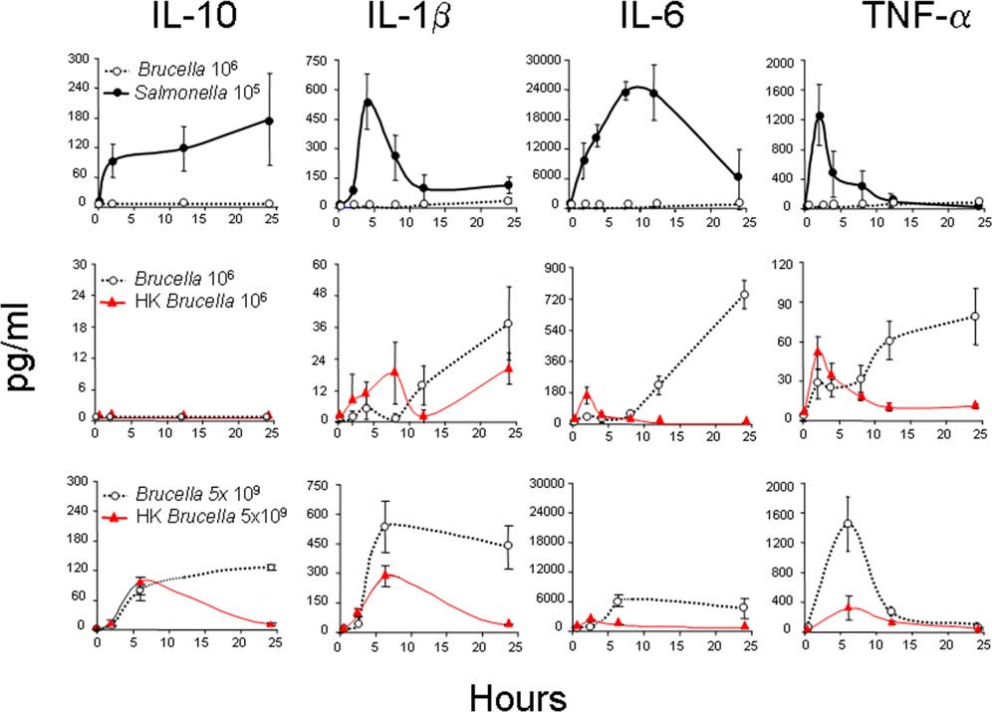
*B. abortus* infections induce a blunted cytokine response. The levels of IL-10, IL-1β, IL-6 and TNF-α were determined in the sera of Balb/c mice (6 per group) intraperitoneally infected with 10^6^ CFU of *B. abortus* 2308, 10^5^ CFU of *S. typhimurium* or injected with 10^6^ CFU of HK-*B. abortus* (upper and center panels). Alternatively, mice were infected with 5×10^9^ CFU of *B. abortus* 2308, or injected with 5×10^9^ of HK-*B. abortus*. Untreated or intraperitoneally injected with 0.1 ml PBS control groups displayed negligible quantities of cytokines (not shown). Notice that the scales of the graphics between the upper and center panels differ in at least one order of magnitude.

Although it has been shown that live- and killed-*Brucella* induce proinflammatory cytokines [8], [11], [13], [21], [33], [34], the *in vivo* levels and kinetics at early times have not been contrasted with those in other infections. As expected, *S. typhimurium* induced a fast increase in TNF-α, IL-1β and IL-6 levels that reached their maximum 2, 4 and 10 h post inoculation, respectively, and then decreased rapidly (Figure 5 upper panel). Concomitantly, the levels of anti-inflammatory IL-10 increased steadily up to at least 24 h of infection. In contrast, the levels of these four cytokines were comparatively insignificant after infection with *B. abortus* (Figure 5 upper panel). This markedly lower response was not linked to an active interference by the live bacteria because inocula of HK-*B. abortus* did not increase the cytokine levels (Figure 5 upper and center panels). It is important to note that, in this and subsequent experiments, HK-bacteria were not washed after killing because it is known that heat disrupts *Brucella* cell envelopes and exposes large amounts of *Br*LPS, BLPs, peptidoglycan, DNA and other molecules [35] as a rule recognized by innate immunity. Although both HK- and live-*B. abortus* displayed effects much lower and widely different from those of *S. typhimurium*, they did not induce identical activities, as demonstrated when the scale of the graphs are modified to portray more resolution (Figure 5 center panel). Live-*B. abortus* stimulated TNF-α, IL-1β and IL-6 biphasic responses with steady increases after 8 h post inoculation, whereas HK-*B. abortus* induced a response with a different kinetics and lower amounts of cytokines after 10 h. Only when mice were infected with a very high dose of *Brucella* (5×10^9 ^CFU), the levels of cytokines approached those induced by *Salmonella* (Figure 5 lower panel). Similarly, 5×10^9^ of HK-*B. abortus* induced a modest increase in cytokines (Figure 5 lower panel) and mild signs of endotoxemia, but not lethality, probably due to the lower levels of TNF-α generated. Moreover, the cytokine profiles were different from those induced by live-bacteria. Since we were able to overcome the low cytokine response by using very large inocula, our results demonstrated that the low levels of proinflammatory cytokines induced by *B. abortus* infection were not connected to inhibitory mechanisms exerted by the infecting bacteria.

### *B. abortus* molecules putatively bearing PAMPs do not inhibit cytokine responses and are low activators

**Figure 6:**
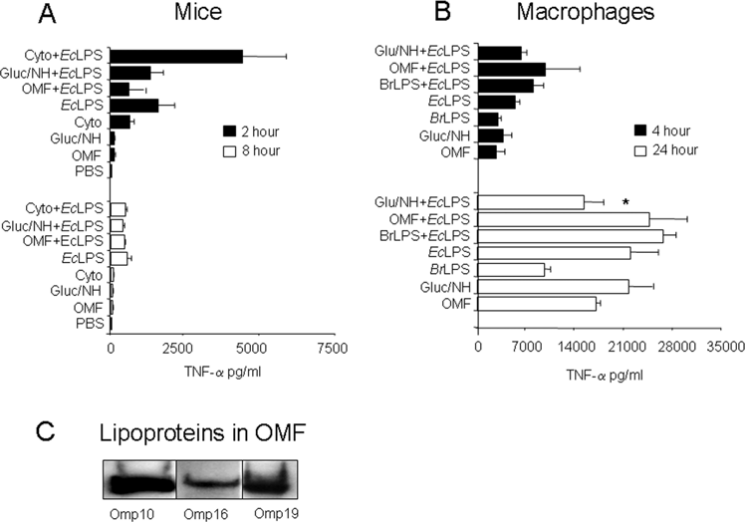
*B. abortus* PAMP-bearing molecules and extracts do not block the generation of TNF-α *in vivo* and *in vitro*. (A) Balb/c mice (10 per group) were intraperitoneally. injected with 50 µg/0.05ml PBS of each of the different *B. abortus* preparations described in Table 1, or with 0.05 ml PBS alone. Then, halve of the mice from each group were intraperitoneally injected with 5 µg/0.05 ml PBS of *Ec*LPS, and the other halve with 0.05 PBS alone, and TNF-α levels determined in sera at 2 and 8 hours after the last injection. (B) RAW264.7 macrophages were treated with 50 µg/well with each of the various preparations described in Table 1. After 30 min, halve of the cultures were challenged with 5 µg/well of *Ec*LPS and the levels of TNF-α determined from culture supernatants at 4 and 24 hours. (C) Omp10, Omp16 and Omp19 lipoproteins in *Brucella* OMF revealed by Western blots with the respective monoclonal antibodies. Value of p<0.05 (*) is indicated.

To test whether the *Brucella* molecules that putatively bear PAMPs hampered activation or, on the other hand, were just poorly detected, we performed two experiments. First, we challenged mice and macrophages with high concentrations of a collection of *B. abortus* fractions containing those molecules (Table 1), and measured the TNF-α levels (Figure 6). Second, we injected *Escherichia coli* LPS (*Ec*LPS) alone or after the respective *B. abortus* fractions and compared the TNF-α levels with those measured in the first experiment. All *B. abortus* fractions induced very low levels of TNF-α in mice after 2 or 8 hours, even at high concentrations (50 µg/mouse) but none of them inhibited the generation of this cytokine after activation with *Ec*LPS. Similarly, only high concentrations of the *B. abortus* preparations (50 µg/ml) generated TNF-α in macrophages and no preparation inhibited a subsequent activation by *Ec*LPS. Concentrations lower that 10 µg/mouse or 10 µg/well of the *B. abortus* fractions induced very low or undetectable levels of cytokines. On the contrary, 0.05–5 µg/well of *Ec*LPS induced significant levels of TNF-α (not shown). Similarly, very low to undetectable levels of the inhibitory cytokine IL-10 were observed in macrophages treated with the *B. abortus* fractions. This was in contrast to *Ec*LPS, which induced significant amounts of this cytokine after 24 h (not shown). None of the *Brucella* fractions was toxic for mice or macrophages. These experiments demonstrate that, in addition of being low activators, the *Brucella* molecules that putatively bear PAMPs (Table 1) do not inhibit the generation of TNF-α *in vivo* or *in vitro*.

**Table 1:** Characteristics of the PAMP-bearing preparations used in this study for stimulating mice and macrophages.

Preparation	Description
*Br*LPS	*B. abortus* LPS possesses an O-amphypatic chain homopolymer composed of N-formyl-perosamine subunits, a core oligosaccharide devoid of negative charges, and a lipid A backbone of two subunits of diaminoglucose substituted with long chain (up to 30 C atoms) of hydroxylated and unsaturated fatty acids. The preparation contains 85% LPS, 11% NH, 1.5% protein and 2.5 % of other components. *B. abortus* LPS mutants are attenuated. [3], [4], [20], [56], [79].
*Ec*LPS	*E. coli* LPS is a classic endotoxic preparation composed of hydrophylic O chain, highly negative charged core oligosaccharide and a canonical lipid A composed of two subunits of glucosamine substituted with short chain (<16 C atoms) hydroxylated and saturated fatty acids. This preparation contains of 87% LPS, 2% protein and 11% other components [3].
Gluc/NH	Preparation NH2 composed of 65% of cyclic beta-1,2-glucan substituted with anionic succinyl residues, 30% NH, and 5% of other components. *B. abortus* mutants in cyclic beta-1,2-glucan or devoid of O-chain derived polysaccharides, including NH are attenuated [79].
OMF	Outer membrane fragments composed of 42% *Br*LPS, 26% NH, 18% proteins, and 10% phospholipids and ornithine-containing lipids. Proteins include 60 periplasmic proteins and 25 OM proteins, from which 10 are BLPs, including Omp10, Omp16 and Omp19. *B. abortus* mutants in several Omps, and BLPs are attenuated [19], [35], [58].
Cyto	This preparation is 96% protein from which 65% are cytoplasmic, 25% periplasmic, 5% membrane associated and 1% membrane. A significant proportion of the cytoplasmic and periplasmic proteins are devoted to folding, sorting, degradation and transport functions. BLPs, LPS, NH or beta-1, 2 cyclic glucans were not detected in this preparation [35], [79].
HK-*B. abortus*	Heat killed *B. abortus* was prepared by boiling *B. abortus* 2308 in pyrogen-free PBS for 10 minutes without further washing the bacterial debris. This preparation includes exposed peptidoglycan, nucleic acids, and mixtures of membrane components.

### Replicating intracellular *B. abortus* are protected from macrophage activation

**Figure 7:**
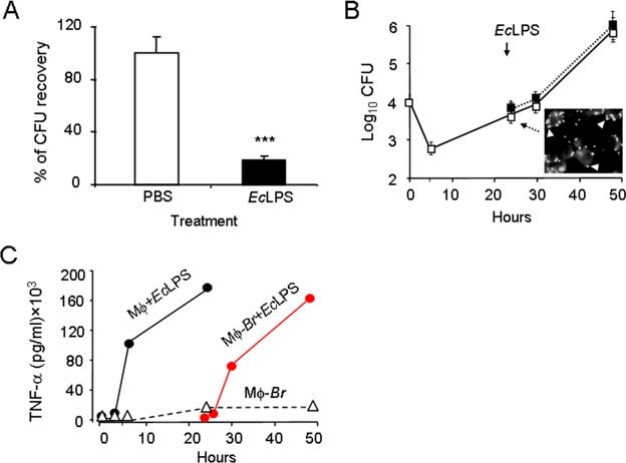
Macrophages activated before infection are significantly more brucellacidal than macrophages activated after infection. (A) Naïve or activated RAW264.7 macrophages with *Ec*LPS 0.5 µg/well 15 h prior to infection, were infected with *B. abortus* 2308 at 10±5 bacteria/cell and the microbicidal function estimated after 2 h post inoculation. Under these conditions no significant cytotoxicity was recorded in these phagocytic cells. (B) Naïve macrophages were infected at rate of 10±5 bacteria/cell and bacteria replication estimated; after 24 h, half of the infected macrophages were activated with 0.5 µg/well *EcLPS* (black squares) and the rest of the cells treated with PBS (white squares) and bacterial replication followed until 48 h post inoculation. Immunofluorescence of replicating *Brucella* at 24 h of infection (arrows) is shown in the inserted figure. (C) TNF-α measured in the supernatants of non-infected *Ec*LPS 0.5 µg/well activated macrophages (black circles), non-activated *B. abortus* infected cells (white triangles), or *Ec*LPS activated cells after 24 h of *B. abortus* infection (red circles). Value of p<0.0005 (***) is indicated.

It has been proposed that activated macrophages are the primary source for *B. abortus* elimination in the infected host [29], [33]. However, our *in vivo* observations suggested that macrophage activation could be both delayed with respect to the onset of *B. abortus* infection and insufficient to lead to an effective control of the pathogen. To study this hypothesis, we first compared the brucellacidal activity of non-activated macrophages and macrophages exogenously activated before or during infection. Macrophages were treated or not with *Ec*LPS, then inoculated with *B. abortus* and CFU determined 2 h later. The results showed that macrophages pretreated with *Ec*LPS were considerably more brucellacidal than naive macrophages (Figure 7 A), and that treatment did not increase the proportion of cell death as compare to the infected controls without *Ec*LPS treatment (not shown). Then, naïve macrophages were infected with *B. abortus,* incubated for 24 h until intracellular replication started. At this time, they were treated with *Ec*LPS or left untreated. As shown in Figure 7 B, *Brucella* replication proceeded unaltered in the macrophages treated with *Ec*LPS at 24 h of infection. In all these experiments, macrophage activation was not endogenously inhibited by the intracellular bacteria, as demonstrated by the production of similar TNF-α levels in infected and non-infected macrophages upon *Ec*LPS stimulation (Figure 7 C). Altogether, these results established that previously activated macrophages display higher brucellacidal activity than naïve ones. Moreover, they demonstrate that *B. abortus* infected macrophages are not refractory to further activation and also that such an activation does not lead to the elimination of replicating *Brucella.*

### TLRs barely respond to high levels of *Brucella* molecules putatively bearing PAMPs and do not influence bacterial replication

**Figure 8:**
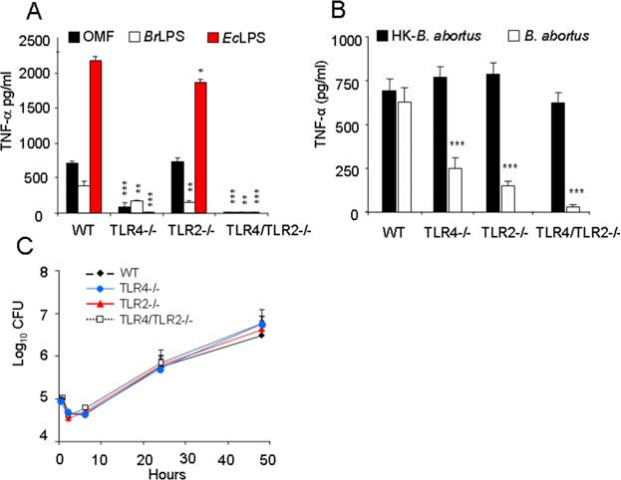
*Brucella* intracellular replication is TLR4 and TLR2 independent but TNF-α production is TLR4 and TLR2 dependent. (A) BM macrophages from WT, TLR4-/-, TLR2-/- and TLR4/TLR2-/- C57Bl/6 mice were inoculated with 50 µg/well of *B. abortus* OMF, 50 µg/well of *BrLPS* or 0.1 µg/well of *Ec*LPS (A) and TNF-α measured after 24 h. (B) BM macrophages from WT, TLR4-/-. TLR2-/- and TLR4/TLR2-/- C57Bl/6 mice were infected with *B. abortus* 2308 (infection rate of 10±5 bacteria/cell) or treated with 10 HK-*B. abortus*/cell and TNF-α measured after 48 h. (C) BM macrophages were infected as in “(B)” and bacterial replication determined as the number of CFU during different periods. Values of p<0.05 (*), p<0.005 (**) and p<0.0005 (***) are indicated.

The lack of macrophage activation induced by *Brucella* could be explained by an insufficient triggering of PAMP receptors. To test this, we measured the production of TNF-α by bone marrow (BM) macrophages of WT, TLR4-/- TLR2-/- and TLR4/TLR2-/- mice upon stimulation with bacterial products, live- and HK- *B. abortus* (Figure 8). The levels of TNF-α induced by the *Brucella* OM fragments (OMF) or *Br*LPS at 50 µg/ml were lower than that induced by *Ec*LPS at 0.1 µg/ml (Figure 8 A). In spite of this, the recognition of purified *Br*LPS and OMF depended on TLR (Figure 8 A). Recognition of OMF, a material that contains high quantities of *Br*LPS and several BLPs (Table 1 and Figure 6 C), did not depend on TLR2; however, the absence of both, TLR4 and TLR2, abolished the response. In a second set of experiments, we compared the TNF-α induction in macrophages inoculated with live- and unwashed HK- *B. abortus.* TLR KOs but not WT macrophages generated differential and lower levels of TNF-α when infected than when inoculated with unwashed HK-*B. abortus* (Figure 8 B), demonstrating *in vitro* dependence on TLR4 and TLR2 by live- but not by HK-bacteria. As expected, the TNF-α levels induced by *Brucella* were at least 3 times lower than those generated in WT macrophages stimulated with *Ec*LPS (Figure 8 panels A and B). These results prompted us to investigate the multiplication of *Brucella* in macrophages deficient for these receptors. In agreement with our previous observations of *in vivo* infections in TLR KO mice [11], the replication of *B. abortus* in TLR4-/-, TLR2-/- and TLR4/TLR2-/- macrophages did not differ from those in WT (Figure 8 C). Therefore, whereas TNF-α secretion in cultured macrophages infected with *B. abortus* seems to depend somewhat on TLR2 and TLR4, signaling by these receptors does not affect the intracellular replication of this pathogen.

### *B. abortus* intracellular replication has no cytotoxic effects

**Figure 9:**
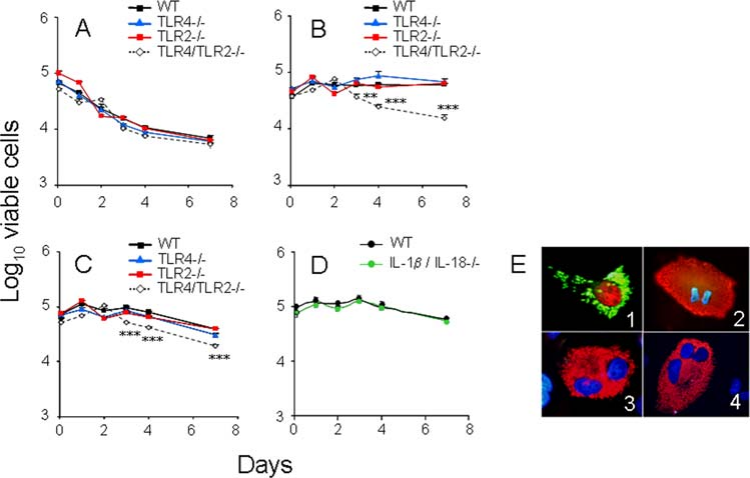
*Brucella* is not cytotoxic for macrophages and HeLa cells. (A) Survival rate of uninfected WT, TLR4-/-, TLR2-/- and TLR4/TLR2-/- BM macrophages from C57Bl/6 mice was followed using MTT assay for seven days. (B) Survival of macrophages infected with *B. abortus* S19 at MOI of 50. (C) Survival of macrophages treated with 50 µg/ml of HK-*B. abortus* S19. (D) Survival of WT and IL-1β/IL-18-/- macrophages infected with *B. abortus* S19 (MOI 50). (E) Untreated (panel 1) and CNF treated (panels 2, 3 and 4) HeLa cells were infected with *B. abortus* 2308 at a MOI of 500 and incubated for 48 h. Untreated cells (panel 1) were incubated with BrdU. All cells were processed for immunofluorescence [39] using anti-*Br*LPS antibodies (green in panel 1 and red in panels 2, 3 and 4) or antibodies against BrdU epitope (red in panel 1). CNF treatment inhibits the cytokinesis while not affecting karyokinesis resulting in the generation heavily infected cells during the mitotic cycle (panel 2, cell in anaphase), binucleated cells (panel 3) or multinucleated cells (panel 4). Values of p<0.005 (**) and p<0.0005 (***) are indicated.

*Salmonella* is cytotoxic for BM macrophages in a TLR2 and/or TLR4-independent manner [36]. In contrast, *Brucella* replicates extensively within macrophages and inhibit apoptosis in human monocytes [18], [37]. Therefore, we determined the survival of WT and different TLR KO macrophages infected with *B. abortus*. The viability of uninfected macrophages steadily decreased during 7 days of culture and there were no significant differences in the death rate among the various types of cells, demonstrating that the TLR KO macrophages do not have a generalized survival deficiency (Figure 9 A). The viability of TLR4/TLR2-/- macrophages infected with *B. abortus* also decreased, although not at the same rate as the uninfected controls (Figure 9 B). On the contrary, no decrease in the viability was detected in *B. abortus*-infected WT, TLR4 -/- and TLR2 -/- macrophages. Similar results were obtained with HK-*Brucella,* suggesting that steady replication within macrophages was not necessary to promote survival (Figure 9 C). Since the IL-1β and IL-18 receptors signal through the MyD88 molecule like TLRs, we tested if IL-1β and IL-18 signaling contributed to macrophage survival after *B. abortus* infection. As shown in Figure 9 D, the decrease in viability was also suppressed in *Brucella* infected IL-1β/IL-18-/- macrophages. These results indicate that *B. abortus,* in addition to its lack of toxicity for BM macrophages, this bacterium was able to promote the survival of these cells, even in the absence of signaling by relevant TLR or cytokine receptors.

Epithelial cells are decisive host cells for the establishment of brucellosis [38]. In order to determine if extensive replication of *Brucella* induced cell death through toxic effects or apoptosis, we infected naïve and *E. coli* cytotoxic necrotizing factor (CNF)-treated (in order to avoid cytokinesis) HeLa cells and monitored the cell cycle. As expected [39], extensively infected HeLa cells were able to synthesize DNA, condense chromosomes and perform several cycles of nuclear division, demonstrating that even large amounts of intracellular *Brucella* not induce toxicity in these cells (Figure 9 E).

### *B. abortus* replication and immunity *in vivo* are not dependent on TLR4

**Figure 10:**
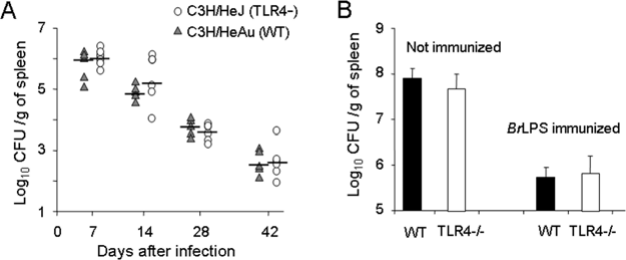
*B. abortus* replicates in naïve and *Br*LPS vaccinated TLR4 deficient mice (A) TLR4 deficient mutant C3H/HeJ and the WT counterpart C3H/HeAu mice were infected with 10^6 ^CFU *B. abortus* S19 and the number of replicating bacteria counted from the spleen at different time periods (5 mice per group). (B) WT and TLR4-/- C57Bl/6 mice were injected with PBS (5 mice per group) or intraperitoneally immunized with *Br*LPS (5 mice per group) and after two weeks infected with 10^6 ^CFU *B. abortus* S19 and the number of replicating bacteria in the spleen of mice counted at 14 days of infection.

There are coincident results showing that TLR2 is not required for the efficient clearance of either virulent or attenuated *B. abortus in vivo* [8], [11]. On the other hand, it has been described [8] that virulent *B. abortus* increased up to one log in the spleens of TLR4-deficient C3H/HeJ mice as compared to the WT counterparts. However, a hampered response of C3H/HeJ mice to *B. abortus* was not noticed in previous works [9], [10] or more recently in C57BL/6 TLR4-/- mice [11]. Since the use of an attenuated *Brucella* strain more easily reveals a permissive effect connected with macrophage dysfunction [40], we first reexamined the *in vivo* role of TLR4 using C3H/HeJ mice and the cognate WT strain infected with attenuated *B. abortus* S19. We found that *B. abortus* S19 replicated and persisted similarly in C3H/HeJ as in WT mice (Figure 10 A). Assays performed with virulent *B. abortus* 2308 also failed to reveal differences in the course of the infection in these mice (not shown). We also examined the role of TLR4 by immunizing TLR4-/- KO and WT mice with *Br*LPS and challenging them with *B. abortus*. Consistent with the antibody responses induced by *B. abortus* LPS in TLR4 deficient mice [11], no differences in protection levels between TLR4-/- and WT mice were observed (Figure 10 B). These results reinforce the proposal that clearance and development of efficient immunity to *B. abortus* is TLR4-independent [11], despite of the fact that *Br*LPS is TLR4 dependent *in vitro,* although at much higher concentrations than other kind of LPSs [3].

## Discussion

We have evaluated the degree and profiles of the early proinflammatory responses induced by *B. abortus* and several PAMP-bearing molecules in comparison with those evoked by the intracellular pathogen *S. typhimurium*. This comparative approach allowed us to estimate the level at which *Brucella* is able to activate innate immunity at the onset of the infection. It is important to note that the data obtained accurately correlate with the absence of endotoxicity signs, the rarity of leucocytosis and neutrophilia and the almost total absence of coagulopathies in patients with brucellosis [15], [41]. Therefore, our observations are complementary to a significant body of clinical data. The picture that emerges from this study is as follows.

After invading, the brucellae come in contact with humoral mediators and are readily ingested by PMN and macrophages [30], [41], and probably by dendritic cells [42]. At this stage, *Brucella* performs several tasks to avoid immediate destruction: first, it circumvents strong activation of the innate immune system; second, the bacterium withstands the direct action of complement and other bactericidal substances; third, it resists and evades the action of professional phagocytes, such as PMN and macrophages; finally, *Brucella* maintains the host cells alive in order to establish long lasting infections.

The first task is accomplished through a negligible induction of proactive plasma inducers of inflammation, insignificant levels of complement fixation and delayed and low levels of cytokines and chemokines, as demonstrated here and in other works [3], [11], [43]. These effects should be connected to the low recruitment of leukocytes at early times and the weak brucellacidal action of PMN. Despite these low-activating properties of *Brucella*, it was striking that the course of infection was unaffected in mice depleted of granulocytes because PMN kill these bacteria at significant higher rates than macrophages [18], [31]. The brucellacidal activity of PMN is promoted by opsonization with normal sera [44] but it seems that the negligible complement binding displayed by *B. abortus* (Figure 4 A) which is related to the poor complement binding of its LPS [4] would be a limiting factor. It is noteworthy that the lack of role of PMN parallels that of NK cells in the sense that they are not crucial for the control of *B. abortus* at early times of infection [21], at least in the murine model. A possible general consequence of these observations is that PMN, the foremost cells of innate immunity, may serve as carriers spreading viable *B. abortus* in the body as proposed by Spink more than 50 years ago [41].

At first glance, these results are in apparent disagreement with previous studies that reported PMN infiltration in *B. abortus* infected organs [29] and increased and reduced bacterial replication in mice depleted of TNF-α or IL-10, respectively [45], [46]. A close examination shows, however, that most of the phenomena described in those studies correspond to late times, once adaptive immunity has initiated or been established [47], and that the cellular infiltrates in the organs are predominately mononuclear [29]. It is also significant that the ability of macrophages to control intracellular *B. abortus* is affected by IL-10 only at very high concentrations [45], and that IL-6 and TNF-α have no major effect [33], in contrast to INF-γ which is the key cytokine in the control of brucellosis [21]. Therefore, our observations at the onset of *B. abortus* infection are not in contradiction with those made once the adaptive immunity has developed.

Activated macrophages control *Brucella* efficiently, an effect noted even before the term “activated macrophage” was coined [22]. Since then, it has remained an unsolved problem how *Brucella* is able to persist in the infected host despite macrophage activation and a patently active and long lasting adaptive immunity. Furthermore, it has been noted that elimination of NADPH oxidase or nitric oxide synthase activity in mice does not affect the recovery of *B. abortus* in these animals [48], suggesting that these and possibly other crucial functions of activated cells are not relevant in brucellosis. Therefore, a very significant observation was that, once established in naïve macrophages, subsequent activation did not lead to *Brucella* clearance. It has been also shown that virulent *B. abortus* induces less macrophage activation than attenuated strains, as estimated by *in situ* esterase staining of mononuclear cells *in vivo* [22].

Live *Brucella* does not release cytotoxic substances [16], [49] and it seems to prolong the survival of macrophages independently of TLR4, TLR2, IL-1β or IL-18. These properties enable *Brucella* to replicate without generating obvious cell damage, a characteristic that may be essential for establishing long lasting chronic infections. Some dependence, however, was observed when both TLRs were absent, suggesting that signaling by at least one of these receptors is required to prologue survival. Similarly, *Brucella* infected epithelial cells are able to cycle and consequently remain as reservoirs for the bacteria *in vivo*. Dendritic cells have been shown to sustain *Brucella* replication [42] and thus may participate in immunity or serve as reservoirs, a hypothesis that deserves experimental testing. Other cells such as lymphocytes, which are crucial for mounting an efficient adaptive immunity at later times [21], may not play a significant role at the onset of the infection. It seems, therefore, that a crucial step in *Brucella* pathogenesis is to sneak to its replicating niche without cell activation or cell cycle interruption. Once there, macrophages are unable to destroy intracellular replicating *Brucella,* not because they are refractory to activation, but rather because their niche becomes unsuitable for fusion with lysosomes. Finally, the apoptosis inhibition [37] may be also linked to the absence of caspases mediating proinflammatory activation.

It is not known whether *Brucella* containing vacuoles fail to fuse with lysosomes because they are part of the endoplasmic reticulum, because these vacuoles are modified in such a way that they do not have the appropriate docking molecules, or both. In this connection, the lack of significant involvement of TLR4 and TLR2 in the initial steps of *B. abortus* infection is remarkable. It has been demonstrated that, whereas early involvement of TLR4 and TLR2 promotes an inducible mode of phagocytosis characterized by a rapid fusion with lysosomes, their absence allows constitutive and slower maturation of the phagocyted particles [50]. Based on the biogenesis of *B. abortus* containing vacuoles in macrophages [18], the slow and low modulation of TLRs in *B. abortus* infected cells [11] and the unaltered replication profiles displayed in TLR4, TLR2 and TLR4/2 KO phagocytes (Figure 8 B), we propose that maturation of the *Brucella* containing phagosome initially follows the constitutive pathway which is then diverted by the activity of *Brucella* factors. Indeed, the β-1,2-glucans and VirB system have been demonstrated to hamper lysosomal fusion and redirect *Brucella* to its replicating niche, once the bacterium has invaded and localized in early vacuoles [17], [18].

The absence of a TLR4 effect on *Brucella* replication in macrophages is in agreement with the results observed in the C3H/HeJ mice, here and in other works [9], [10], [51], and with our previous experiments in TLR4-/- mice [11], but in apparent contradiction with others [8], [12]. Although we do not know the reasons for these discrepancies, there are several considerations that can be made. First, the *Brucella* and mice strains used (e.g. *B. abortus* versus *B. melitensis*; *Brucella* resistant versus sensitive mice) as well as the bacterial doses and experimental settings, differ among the various works. Second, the differences in CFU observed at early times between TLR4 KO and WT mice, although statistically demonstrable display low significance and it is manifested at early times but not at later times [12], suggesting low influence of this TLR in the course of *Brucella* infection. It is also significant that TLR4-/-, TLR4/2-/- and C3H/HeJ mice infected with *Brucella* or immunized with *Br*LPS generate strong anti-LPS antibody responses [11], [52], and that these antibodies were protective in TLR4-/- mice (Figure 10), suggesting low relevance of TLR4 for immunity against *Brucella*. These observations point out that TLR4, a conspicuous LPS cell receptor of the innate immune system, is not important for mounting an efficient and protective immune response against *Brucella* as it is the case in other gram negative infections [53]. However, Myd88, which is the adaptor molecule for several TLRs and interleukin receptors, is clearly required for the control of *Brucella* replication in mice [11], [12], suggesting that some signaling through receptors that use Myd88 is required to control brucellosis, mainly once adaptive immunity has taken place.

The ability of *Brucella* to behave as a stealthy parasite seems to be connected to physiological and structural features and not to classical virulence factors specifically designed to foil the immune system [16]. It is clear that early innate immunity detection failed mostly because of both the absence or reduced number of molecules with canonical PAMPs and the lack of cytotoxic substances generated by *B. abortus*. Indeed, *Brucella* is devoid of surface structures such as capsules, fimbriae and pili, structures that are all conspicuous in many soil living *Brucella* relatives [54]. In addition, *Br*LPS poorly binds cationic bactericidal peptides [6], [55] and, although it induces TLR4-dependent cytokine responses, its bioactivity is markedly lower than that of canonical LPSs, as demonstrated here and in previous works [3]–[5], [11], [20]. These differences are largely accounted for by the relative reduction of anionic groups in the core oligosaccharide, diaminoglucose backbone and the presence of long and very long acyl chains in amide and acyloxyacyl linkages in the lipid A [56]. These features may also hamper its interaction with coupling molecules such lipid binding proteins, CD14 and MD-2, as suggested elsewhere [57].

In addition to LPS, there are other PAMP bearing molecules in the gram-negative OM. Based on the analysis of the activity of *B. abortus* BLPs purified from recombinant *E. coli*, it has been proposed that they are key elements triggering proinflammatory responses [14]. In our work, the cytokine levels induced *in vitro* and *in vivo* by live- and HK-*Brucella* as well as by OMF are far lower than those induced by *Salmonella*. Since OMF contain significant quantities of the three major *B. abortus* BLPs (Figure 6 C), our results indicate that they are poor inducers of innate immunity, at least in the context of the whole bacterium or in OMF. It has been noted that triggering of TLR2 by lipopeptides and lipoproteins depends dramatically on the type of fatty acids substituting these molecules and to less extent on their amino acid composition [58]. Thus, one possible explanation for the differences between our observations and those with cloned *B. abortus* BLPs is that the latter carry the acylation pattern characteristic of *E. coli*. The chain length of the bound fatty acids is larger in *Brucella* than in *Enterobacteriaceae* [59] and, in an early study on the trypsin fragment of a *Brucella* BLP, one of us reported that the fatty acids differed considerably from those in the peptidoglycan-linked *E. coli* lipoprotein [60]. Although structural studies are necessary for a definite conclusion, the equivalent response of WT and TLR2-/- as well as the lower response of TLR4-/- BM macrophages treated with OMF support the hypothesis that *Brucella* BLPs display a structure with reduced or altered PAMP. An additional factor that could account for the differences between *B. abortus* and *Salmonella* may lay in their BLP content. Proteomic studies have identified 15 different BLPs in *B. abortus* OMF [61], a number that contrasts with the more than 100 putative BLPs present in *Salmonella* genomes [62]. Cell envelope ornithine-containing lipids from several bacteria have also been shown to be strong inducers of cytokines and prostaglandins [63]. However, the structures of *Brucella* ornithine-containing lipids (also present in the OMF) differ from those of *Achromobacter, Bordetella* and *Flavobacterium* in at least the type of fatty acids [59], [63]. Concerning flagella, although the brucellae are non-motile, this structure can be expressed on the surface of these bacteria [64]. However, *Brucella* flagellin, the putative cognate PAMP molecule for TLR5, displays an amino acid sequence not recognized by this receptor [65].

The cytokine profiles and TLR dependence observed *in vivo* or in cultured cells with live- and killed-bacteria or their isolated PAMPs display a good correlation in many pathogens, including *Salmonella* [66], [67]. This was not the case with *B. abortus*. In contrast to OMF (devoid of DNA and peptidoglycan), unwashed HK-*B. abortus* induced cytokine profiles that were not dependent on TLR4 and TLR2. It is likely that TLR9 and NOD-like receptors which are the cognate receptors for *Brucella* DNA [12] and may be for its canonical peptidoglycan [60], respectively, are the responsible receptors for inducing these TLR4 and TLR2 independent responses *in vitro*. It may be also that TLR9 [12] and eventually NOD-like receptors functioning intracellularly, are the relevant TLRs for controlling *Brucella* infection through specialized dendritic cells acting in concert with other cells for generating IFN-γ [12]. However, this still does not explain the negligible levels of proinflammatory cytokines *in vivo*, mainly when PAMPs such as DNA and peptidoglycan are readily accessible in HK-*Brucella.* Why did HK-*B. abortus* induce a low level of cytokines *in vivo* and why did the live bacteria show TLR4 and TLR2 dependence for cytokine release in macrophages and no dependence on these TLRs during replication *in vivo* [11] or *in vitro* (Figure 8 C)? This is surprising because a number of *B. abortus* are killed by macrophages during the first hours of infection [18], [68] without significant activation of the infected cells, as demonstrated here. All these results indicate that the availability of *Brucella* PAMPs within infected cells or elsewhere in the host is not straightforward and that those interpretations based on the interaction between *Brucella* molecules putatively bearing PAMPs and cell receptors require careful attention.

It is tempting to speculate that the stealthy strategy of *Brucella* corresponds to an evolutionary path that has been followed by several pathogenic α*-Proteobacteria* by taking advantage of a common structural heritage. For instance, *Bartonella*, another intracellular parasite inducing long lasting infections in mammals, shares with *Brucella* the chemical and biological characteristics of its lipid A, core oligosaccharide, major fatty acids, low number of BLPs, putative enzymes to built ornithine-lipids, and flagella not recognized by TLR5 [62], [65], [69], [70]. The brucellae, and probably other pathogenic α-*Proteobacteria* have developed furtive characteristics by eradicating, modifying or hiding otherwise moderately active PAMP-bearing molecules, to the point that practically not recognized by the corresponding receptors. In the case of *Brucella*, this is complemented by the maintenance of β-1,2-glucans, type IV secretion apparatus, *Br*LPS and sensing and regulatory systems that allow to reach a safe intracellular niche before an effective immune response is developed [17]–[21].

## Materials and Methods

### Bacterial strains, fractions and biological reagents

*Salmonella enterica* sv. Typhimurium strain SL1344, virulent *B. abortus* 2308 and attenuated *B. abortus* S19 were grown as described [54], [71]. Description and characterization of *Br*LPS, *Ec*LPS, OMF, β-1,2-glucans, native hapten polysaccharide (NH), cytoplasmic fractions and HK-*B. abortus* are described in Table 1. The characteristics of bactericidal cationic peptide p-EM2, PMN-extracts, CNF and anti-*Brucella* and anti-*S. typhimurium* antibodies, have been described previously [6], [39], [55], [72]. Enzyme linked immunosorbent assay (ELISA) kits for cytokine determination were from BD Biosciences (San Diego, CA). Monoclonal antibody RB6-8C5 against murine granulocytes was a gift from Bärbel Raupach, MPIIB, Berlin, Germany. Monoclonal antibodies against Omp10, Omp16 and Omp19 were a gift from Axel Cloeckaert, Unité BioAgresseurs, Santé et Environnement, INRA, France.

### Experimental animals

The characteristics, source and maintenance of Balb/c, C3H/HeJ, C3H/HeAu, C57BL/6 and the KO counterparts TLR4-/-, TLR2-/-, TLR4/TLR2-/- and IL-1β/IL-18-/- mice have been described previously [11], [52]. Wistar rats were maintained in the animal facility of the Veterinary School of the National University, Costa Rica. All animals were handled and sacrificed according to the guidelines of the “Comité Institucional para el Cuido y Uso de los Animales of the Universidad de Costa Rica”, and in agreement with the corresponding law “Ley de Bienestar de los animals N°7451″ of Costa Rica. Mice were infected by the intraperitoneal route with 0.1 ml of bacteria in pyrogen-free sterile PBS.

### Generation of neutropenic mice

Balb/c mice were intraperitoneally injected with 100 µg of RB6-8C5 antibody, 36 h before infection. Then mice were infected with 10^6^ *B. abortus* or 10^5^ *S. typhimurium*. Controls were injected with sterile PBS. Thereafter, mice were injected with 100 µg every two days (one injection for *S. typhimurium* and 3 injections for *B. abortus*) until sacrificed. One single intraperitoneal injection of 100 µg of RB6 antibody resulted in PMN depletion for at least 3 days. Under these circumstances the blood of mice bled from the tail, did not demonstrate granulocytes during the course of experiments, as judged by Giemsa-Wright staining of the smears. Neither circulating nor resident populations of macrophages, lymphocytes or other resident cells were affected by this treatment. On day 3, mice infected with *S. typhimurium* and the respective controls were sacrificed. *B. abortus* infected mice and the respective controls were sacrificed on days 7 and 14. Bacterial colonization was determined in spleens of mice collected at the indicated times, then weighed and homogenized in 1 ml sterile PBS. Serial dilutions were plated on Luria agar plates for *S. typhimurium* and trypticase soy agar for *B. abortus* and CFU per g of spleen calculated.

### Macrophage, HeLa and PMN cell cultures

Murine RAW264.7 (ATCC TIB-71), HeLa (ATCC CCL-2) cells and BM-derived macrophages from C57BL/6 and the KO counterparts TLR4-/-, TLR2-/-, and TLR4/TLR2-/- mice were prepared, cultured and infected following previous protocols [11], [18], [39]. Fresh preparations or PMN were obtained from air pouches of Wistar rats (150-200 g) as described by Fukura and Tsurufuji [73]. Human PMN were purified from heparinized blood as described elsewhere [74]. The human cell preparations and rat exudates were composed from 95-99 % PMN per nucleated cells. Erythrocyte contamination was less that 5% of the packed cells. PMN preparations were maintained at 4°C in PBS, and used within the first h after extraction.

### Cell infections

Infections of BM and RAW264.7 macrophages and HeLa cells were carried out as described [11], [36], [39]. Briefly, plates containing 5×10^5^ RAW264.7 or 2×10^5^ BM macrophages were inoculated at the ratio of 200 or 50 *B. abortus* per cell, respectively. For *S. typhimurium*, a rate of infection of 2 bacteria per cell was used. At these multiplicities, similar numbers of *B. abortus* and *S. typhimurium* infected cultured macrophages as determined by immunoflurescence. PMN infections were performed as follows: suspensions of 10^6^ rat PMN in 0.5 ml of PBS supplemented with 0.2 mM CaCl_2_, 5 mM MgCl_2_ and 10% of human serum, were infected with *B. abortus* or *S. typhimurium* at multiplicity of infection (MOI) ranging from 5 to 100 bacteria per cell, and the mixture incubated for 15 minutes at 37°C under mild rotation. PMN/bacteria mixtures were centrifuged at 2000 *g* to remove non-ingested bacteria. Then, infected PMN were suspended in supplemented PBS in the presence of 10 µg/ml of gentamicin for 30 minutes at room temperature to kill extracellular bacteria. Tubes were centrifuged at 2500 *g* for 5 minutes and cell pellets resuspended in 400 µl of supplemented PBS without gentamicine and incubated at 37°C under mild rotation for additional 45 and 90 minutes. The proportion of infected cells and the number of bacteria per cell was directly determined by direct immunofluorescence using fluorescein isotiocyanate-IgG anti-*Brucella* LPS as described elsewhere [39]. Under these experimental conditions, from 70–85 % of the macrophages were infected at initial times, with 1–3 *B. abortus* per macrophage, while close to 100 % of the PMN were infected with 1–10 bacteria per cell. For macrophages, plates were washed with PBS and cells lysed by adding 0.1% Triton X-100 (Sigma) for 10 minutes. For PMN, samples were centrifuged and the infected cells resuspended 50 µl of PBS and then lysed by adding 200 µl of 0.1% Triton X-100. For determination the number of *B. abortus*, aliquots were plated in tryptic soy agar; for *S. typhimurium* CFU determination, LB agar plates were used.

### Cell functions and immunofluorescence

*In situ* respiratory burst of infected and non-infected rat PMN was estimated by the reduction of nitroblue tetrazolium (NBT, from Sigma) to dark-blue insoluble formazan granules trapped within vacuoles as described [74]. The number of non-infected rat PMN with positive NBT granules was considered as background and subtracted from the total number of infected cells presenting positive granules. Degranulation of rat PMN was estimated by counting in the number of intact cells remaining in a 40× field by phase contrast microscopy. For this, infected and non-infected PMN were incubated at 37°C in supplemented PBS without antibiotics or serum, under mild rotation during different time periods. Macrophage activation was performed on infected and non-infected cells by adding 0.5 µg/ml of *Ec*LPS in the well. Medium samples from infected and uninfected cells were taken at different times and frozen at −20°C until used for TNF-α determination. Procedures for immunofluorescence microscopy have been described elsewhere [39]. For *B. abortus* detection, the fluorescein isotiocyanate-conjugated anti-*Brucella* antibody was directly used. For *S. typhimurium* detection, cells were first incubated with mouse anti-*S. typhimurium* antibody and then with TRITC-conjugated anti-mouse antibody. Counts of cell associated bacteria were performed in at least 100 infected cells.

Toxicity of replicating *Brucella* in cells was estimated in BM macrophages and HeLa cells as previously described [39], [75]. Briefly, 3-(4,5-dimethylthiazolyl-2)-2, 5-diphenyltetrazolium bromide (MTT, from Sigma Chemical Co., St. Louis, Mo) was added to monolayers of BM macrophages at each time point. Then, cells were incubated for 2 h at 37° C, 7% CO2, lysed and the rate of survival estimated by colorant release at the optical density of 570 nm. Two-fold dilutions of cells were freshly seeded and used to make a standard curve. DNA synthesis was estimated by immunofluorescence in 48 h *B. abortus* infected HeLa cells, using 10 µg/ml bromodeoxyuridine (BrdU; Sigma) in combination with monoclonal anti-BrdU (Clone BU-33; Sigma). Nuclei were contrasted with Hoescht stain (Sigma). Replication of *Brucella* in dividing HeLa cells treated with CNF was recorded by immunofluorescence after 48 h.

### Determination of fibrinogen, fibrin dimers, and cytokines

For fibrinogen, fibrin dimers were determined from the plasma of infected mice by the clotting method of Clauss [76], and the semi-quantitative D-Di test® latex agglutination assay (Diagnostica Stago), respectively. The levels of IL-10, IL-1β, IL-6 and TNF-α were estimated by ELISA according to the manufacturer's specifications, in the culture supernatants of macrophages or in the sera of Balb/c mice intraperitoneally infected with *S. typhimurium*, *B. abortus*, or intraperitoneally inoculated with fresh killed HK-*B. abortus*.

### Platelets and leukocyte counts

Balb/c mice from 18 and 20 g were intraperitoneally injected with 10^5^ CFU of *B. abortus* , *S. typhimurium* or 0.1 ml pyrogen-free sterile PBS, and blood from tail collected in heparinized glass capillary tubes at different time points. Alternatively, 5 ml of ice cold PBS were injected in the peritoneal cavity of killed the mice, and the fluids collected with a syringe (from 3.8 to 4.5 ml) from exposed peritoneal cavity [77]. Then fluids were centrifuged and the peritoneal cells resuspended in 0.2 ml of PBS and counted. Four estimating the recruitment of leukocytes sterile in air pouches Balb/c 25–30 g mice, the procedure of García-Ramallo et al. [78] was followed. Briefly, anesthetized animals were subcutaneous injected with 2.5 ml of sterile air under the dorsal skin on day 0 and day 3; three days after, 10^6 ^of *B. abortus* or 10^5^ *S. typhimurium* in 1 ml PBS were injected into the air pouch cavities. Animals were sacrificed at different time points and fluids from the pouches harvested and cells counted. Total leukocytes, PMN, red blood cells and platelets were counted in each sample using an ABX Micros 60 analyzer (ABX Hematologie, France) and confirmed in blood smears. The number of cells recruited in the peritoneum or in air pouches was corrected according to the volume of fluid collected in each animal.

### Determination of complement consumption and bactericidal action of molecules

Complement consumption estimated as the reduction of the hemolytic activity of serum complement incubated with live bacteria, was determined as described elsewhere [77]. Bactericidal action mediated by PMN-extracts and cationic peptide pEM-2 was performed as previously described [55], [72]. For the estimation of complement bactericidal activity the protocol performed previously was followed [20].

### Statistics

Student's t test for was used for determining the statistical significance in the different assays. For bacterial colonization experiments, the Mann-Whitney test was performed accordingly (http://faculty.vassar.edu/lowry/utest.html).

## References

[1] Janeway CA, Medzhitov R (2002). Innate immune recognition.. Annu Rev Immunol.

[2] Pappas G, Akritidis N, Bosilkovski M, Tsianos E (2005). Brucellosis.. N Engl J Med.

[3] Lapaque N, Takeuchi O, Corrales F, Akira S, Moriyón I (2006). Differential inductions of TNF-alpha and IGTP, IIGP by structurally diverse classic and non-classic lipopolysaccharides.. Cell Microbiol.

[4] Moreno E, Berman DT, Boettcher LA (1981). Biological activities of *Brucella abortus* lipopolysaccharides.. Infect Immun.

[5] Rasool O, Freer E, Moreno E, Jarstrand C (1992). Effect of *Brucella abortus* lipopolysaccharides on the oxidative metabolism and enzyme release of neutrophils.. Infect Immun.

[6] Freer E, Moreno E, Moriyón I, Pizarro-Cerda J, Weintraub A (1996). *Brucella*-*Salmonella* lipopolysaccharide chimeras are less permeable to hydrophobic probes and more sensitive to cationic peptides and EDTA than are their native *Brucella* sp counterparts.. J Bacteriol.

[7] Lapaque N, Moriyón I, Moreno E, Gorvel JP (2005). *Brucella* lipopolysaccharide acts as a virulence factor.. Curr Opin Microbiol.

[8] Campos MA, Rosinha GM, Almeida IC, Salgueiro XS, Jarvis BW (2004). Role of Toll-like receptor 4 in induction of cell-mediated immunity and resistance to *Brucella abortus* infection in mice.. Infect Immun.

[9] Cannat A, Serre A (1981). Genetic factors involved in murine susceptibility to experimental brucellosis.. Ann Immunol (Paris).

[10] Phillips M, Pugh GW, Deyoe BL (1989). Duration of strain 2308 infection and immunogenicity of *Brucella abortus* lipopolysaccharide in five strains of mice.. Am J Vet Res.

[11] Weiss DS, Takeda K, Akira S, Zychlinsky A, Moreno E (2005). MyD88, but not toll-like receptors 4 and 2, is required for efficient clearance of *Brucella abortus.*. Infect Immun.

[12] Copin R, De Baetselier P, Carlier Y, Letesson JJ, Muraille E (2007). MyD88-dependent activation of B220-CD11b+LY-6C+ dendritic cells during *Brucella melitensis* infection.. J Immunol..

[13] Huang LY, Aliberti J, Leifer CA, Segal DM, Sher A (2003). Heat-killed *Brucella abortus* induces TNF and IL-12p40 by distinct MyD88-dependent pathways: TNF, unlike IL-12p40 secretion, is Toll-like receptor 2 dependent.. J Immunol.

[14] Giambartolomei GH, Zwerdling A, Cassataro J, Bruno L, Fossati CA (2004). Lipoproteins, not lipopolysaccharide, are the key mediators of the proinflammatory response elicited by heat-killed *Brucella abortus.*. J Immunol.

[15] Ariza J (1999). Brucellosis: an update. The perspective from the Mediterranean basin.. Rev Med Microbiol.

[16] Moreno E, Moriyón I (2002). *Brucella melitensis*: a nasty bug with hidden credentials for virulence.. Proc Natl Acad Sci U S A.

[17] Arellano-Reynoso B, Lapaque N, Salcedo S, Briones G, Ciocchini AE (2005). Cyclic beta-1,2-glucan is a *Brucella* virulence factor required for intracellular survival.. Nat Immunol.

[18] Celli J, de Chastellier C, Franchini DM, Pizarro-Cerda J, Moreno E (2003). *Brucella* evades macrophage killing via VirB-dependent sustained interactions with the endoplasmic reticulum.. J Exp Med.

[19] Guzmán-Verri C, Manterola L, Sola-Landa A, Parra A, Cloeckaert A (2002). The two-component system BvrR/BvrS essential for *Brucella abortus* virulence regulates the expression ofmembrane proteins with counterparts in members of the Rhizobiaceae.. Proc Natl Acad Sci U S A.

[20] Manterola L, Moriyón I, Moreno E, Sola-Landa A, Weiss DS (2005). The lipopolysaccharide of *Brucella abortus* BvrS/BvrR mutants contains lipid A modifications and has higher affinity for bactericidal cationic peptides.. J Bacteriol.

[21] Baldwin C, Goenka R, López-Goñi I, Moriyón I (2004). Host cellular immune responses against *Brucella* spp Evaluated using the mouse model. *Brucella*: Molecular and Cellular Biology Norfolk.

[22] Elberg SS, Schneider P, Fong J (1957). Cross-immunity between *Brucella melitensis* and Mycobacterium tuberculosis; intracellular behavior of *Brucella melitensis* in monocytes from vaccinated animals.. J Exp Med.

[23] Bohme DH, Schneider HA, Lee JM (1959). Some physiopathological parameters of natural resistance to infection in murine salmonellosis.. J Exp Med.

[24] Mastroeni P, Sheppard M (2004). *Salmonella* infections in the mouse model: host resistance factors and *in vivo* dynamics of bacterial spread and distribution in the tissues.. Microbes Infect.

[25] Chen D, Giannopoulos K, Shiels PG, Webster Z, McVey JH (2004). Inhibition of intravascular thrombosis in murine endotoxemia by targeted expression of hirudin and tissue factor pathway inhibitor analogs to activated endothelium.. Blood.

[26] Kwak SH, Wang XQ, He Q, Fang WF, Mitra S (2006). Plasminogen activator inhibitor-1 potentiates LPS-induced neutrophil activation through a JNK-mediated pathway.. Thromb Haemost.

[27] Cheminay C, Chakravortty D, Hensel M (2004). Role of neutrophils in murine salmonellosis.. Infect Immun.

[28] Rajashekara G, Glover DA, Krepps M, Splitter GA (2005). Temporal analysis of pathogenic events in virulent and avirulent *Brucella melitensis* infections.. Cell Microbiol.

[29] Ackermann MR, Cheville NF, Deyoe BL (1988). Bovine ileal dome lymphoepithelial cells: endocytosis and transport of *Brucella abortus* strain 19.. Vet Pathol.

[30] Enright FM, Araya LN, Elzer PH, Rowe GE, Winter AJ (1990). Comparative histopathology in BALB/c mice infected with virulent and attenuated strains of *Brucella abortus*.. Vet Immunol Immunopathol.

[31] Riley LK, Robertson DC (1984). Ingestion and intracellular survival of *Brucella abortus* in human and bovine polymorphonuclear leukocytes.. Infect Immun.

[32] Kossack RE, Guerrant RL, Densen P, Schadelin J, Mandell GL (1981). Diminished neutrophil oxidative metabolism after phagocytosis of virulent *Salmonella typhi.*. Infect Immun.

[33] Jiang X, Baldwin CL (1993). Effects of cytokines on intracellular growth of *Brucella abortus.*. Infect Immun.

[34] Rafiei A, Ardestani SK, Kariminia A, Keyhani A, Mohraz M (2006). Dominant Th1 cytokine production in early onset of human brucellosis followed by switching towards Th2 along prolongation of disease.. J Infect.

[35] Gamazo C, Winter AJ, Moriyón I, Riezu-Boj JI, Blasco JM (1989). Comparative analyses of proteins extracted by hot saline or released spontaneously into outer membrane blebs from field strains of *Brucella ovis* and *Brucella melitensis.*. Infect Immun.

[36] Weiss DS, Raupach B, Takeda K, Akira S, Zychlinsky A (2004). Toll-like receptors are temporally involved in host defense.. J Immunol.

[37] Gross A, Terraza A, Ouahrani-Bettache S, Liautard JP, Dornand J (2000). In vitro *Brucella suis* infection prevents the programmed cell death of human monocytic cells.. Infect Immun.

[38] Kim S, Lee DS, Watanabe K, Furuoka H, Suzuki H (2005). Interferon-gamma promotes abortion due to *Brucella* infection in pregnant mice.. BMC Microbiol.

[39] Chaves-Olarte E, Guzmán-Verri C, Meresse S, Desjardins M, Pizarro-Cerda J (2002). Activation of Rho and Rab GTPases dissociates *Brucella abortus* internalization from intracellular trafficking.. Cell Microbiol.

[40] Pizarro-Cerdá J, Desjardins M, Moreno E, Akira S Gorvel JP (1999). Modulation of endocytosis in nuclear factor IL-6 (-/-) macrophages is responsible for a high susceptibility to inracellualar bacterial infection.. J Immunol.

[41] Spink WW (1956). The nature of brucellosis:.

[42] Billard E, Cazevieille C, Dornand J, Gross A (2005). High susceptibility of human dendritic cells to invasion by the intracellular pathogens *Brucella suis*, *B. abortus*, and *B. melitensis*.. Infect Immun.

[43] Rittig MG, Kaufmann A, Robins A, Shaw B, Sprenger H (2003). Smooth and rough lipopolysaccharide phenotypes of *Brucella* induce different intracellular trafficking and cytokine/chemokine release in human monocytes.. J Leukoc Biol.

[44] Young EJ, Borchert M, Kretzer FL, Musher DM (1985). Phagocytosis and killing of *Brucella* by human polymorphonuclear leukocytes.. J Infect Dis.

[45] Fernandes DM, Baldwin CL (1995). Interleukin-10 downregulates protective immunity to *Brucella abortus.*. Infect Immun.

[46] Zhan Y, Liu Z, Cheers C (1996). Tumor necrosis factor alpha and interleukin-12 contribute to resistance to the intracellular bacterium *Brucella abortus* by different mechanisms.. Infect Immun.

[47] Murphy EA, Parent M, Sathiyaseelan J, Jiang X, Baldwin CL (2001). Immune control of *Brucella abortus* 2308 infections in BALB/c mice.. FEMS Immunol Med Microbiol.

[48] Sun YH, den Hartigh AB, Santos RL, Adams LG, Tsolis RM (2002). virB-mediated survival of *Brucella abortus* in mice and macrophages is independent of a functional inducible nitric oxide synthase or NADPH oxidase in macrophages. Infect.. Immun.

[49] Marchesini MI, Ugalde JE, Czibener C, Comerci DJ, Ugalde RA (2004). N-terminal-capturing screening system for the isolation of *Brucella abortus* genes encoding surface exposed and secreted proteins.. Microb Pathog.

[50] Blander JM, Medzhitov R (2004). Regulation of phagosome maturation by signals from toll-like receptors.. Science.

[51] Pugh GW, Zehr ES, Meador VP, Phillips M, McDonald TJ (1989). Immunologic, histopathologic, and bacteriologic responses of five strains of mice to *Brucella abortus* strain 2308.. Am J Vet Res.

[52] Moreno E, Kurtz RS, Berman DT (1984). Induction of immune and adjuvant immunoglobulin G responses in mice by *Brucella* lipopolysaccharide.. Infect Immun.

[53] Killion JW, Morrison DC (1986). Protection of C3H/HeJ mice from lethal *Salmonella* typhimurium LT2 infection by immunization with lipopolysaccharide-lipid A-associated protein complexes.. Infect Immun.

[54] Moreno E, Moriyón I, Dorkin M, Falkow S, Rosenberg E, Schleifer KH, Stackebrandt E (2001). The genus *Brucella*. The Prokaryotes, Vol 5 (Part 1, section 3.1).

[55] Martínez de Tejada G, Pizarro-Cerdá J, Moreno E, Moriyón I (1995). The outer membranes of *Brucella* spp are resistant to bactericidal cationic peptides.. Infect Immun.

[56] Moriyón I, Gorvel JP (2004). Against gram-negative bacteria: the lipopolysaccharide case. Intracellular pathogens in membrane interactions and vacuole biogenesis.

[57] Dueñas AI, Orduña A, Crespo MS, García-Rodríguez C (2004). Interaction of endotoxins with Toll-like receptor 4 correlates with their endotoxic potential and may explain the proinflammatory effect of *Brucella* spp. LPS.. Int Immunol..

[58] Buwitt-Beckmann U, Heine H, Wiesmuller KH, Jung G, Brock R (2005). Lipopeptide structure determines TLR2 dependent cell activation level.. Febs J.

[59] Thiele OW, Asselineau J, Lacave C (1969). On the fatty acids of *Brucella abortus* and *Brucella melitensis*.. Eur J Biochem.

[60] Gómez-Miguel MJ, Moriyón I (1986). Demonstration of a peptidoglycan-linked lipoprotein and characterization of its trypsin fragment in the outer membrane of *Brucella* spp.. Infect Immun.

[61] Lamontagne J, Butler H, Chaves-Olarte E, Hunter J, Schirm M (2007). Extensive cell envelope modulation is associated with virulence in *Brucella* abortus.. J. Proteom. Res.

[62] Babu MM, Priya ML, Selvan AT, Madera M, Gough J (2006). A database of bacterial lipoproteins (DOLOP) with functional assignments to predicted lipoproteins. J Bacteriol..

[63] Kawai Y, Takasuka N, Inoue K, Akagawa K, Nishijima M (2000). Ornithine-containing lipids stimulate CD14-dependent TNF-alpha production from murine macrophage-like J774.1 and RAW 264.7 cells.. FEMS Immunol Med Microbiol.

[64] Fretin D, Fauconnier A, Köhler S, Halling S, Léonard S (2005). The sheathed flagellum of *Brucella melitensis* is involved in persistence in a murine model of infection.. Cell Microbiol.

[65] Andersen-Nissen E, Smith KD, Strobe KL, Barrett SL, Cookson BT (2005). Evasion of Toll-like receptor 5 by flagellated bacteria.. Proc Natl Acad Sci U S A.

[66] Sha J, Fadl AA, Klimpel GR, Niesel DW, Popov VL (2004). The two murein lipoproteins of *Salmonella enterica* serovar Typhimurium contribute to the virulence of the organism.. Infect Immun.

[67] Lembo A, Kalis C, Kirschning CJ, Mitolo V, Jirillo E (2003). Differential contribution of Toll-like receptors 4 and 2 to the cytokine response to *Salmonella enterica* serovar Typhimurium and *Staphylococcus aureus* in mice.. Infect Immun.

[68] Moreno E, Gorvel JP, López-Goñi I, Moriyón I (2004). Invasion, intracellular trafficking and replication of *Brucella* organisms in professional and non-professional phagocytes. *Brucella*: Molecular and Cellular Biology Norfolk.

[69] Zahringer U, Lindner B, Knirel YA, van den Akker WM, Hiestand R (2004). Structure and biological activity of the short-chain lipopolysaccharide from *Bartonella henselae* ATCC 49882T.. J Biol Chem.

[70] Sander A, Buhler C, Pelz K, von Cramm E, Bredt W (1997). Detection and identification of two *Bartonella henselae* variants in domestic cats in Germany.. J Clin Microbiol.

[71] Guzmán-Verri C, Chaves-Olarte E, von Eichel-Streiber C, López-Goni I, Thelestam M (2001). GTPases of the Rho subfamily are required for *Brucella abortus* internalization in nonprofessional phagocytes: direct activation of Cdc42.. J Biol Chem.

[72] Santamaría C, Larios S, Pizarro-Cerdá J, Gorvel JP, Bokarewa M (2005). Bactericidal and anti-endotoxic properties of short cationic peptides derived from Bothrops asper myotoxin II, a snake venom Lys49 phospholipase A2.. Antimicrob Agents Chemother.

[73] Fukuhara M, Tsurufuji S (1969). The effect of locally injected anti-inflammatory drugs on the carrageenin granuloma in rats.. Biochem Pharmacol.

[74] Coates TD, Beyer LL, Baehner RL, Rose NR, Conway-de-Macario E, Fahey JL, Friedman H, Penn GM (1992). Laboratory evaluation of neutropenia and neutrophil dysfunction. Manual of Clinical Laboratory Immunology Fourth edition.

[75] Weiss DS, Zychlinsky A, Sansonetti P, Zychlinsky A (2002). Methods for studying bacteria-induced host cell death. Molecular and Cellular Microbiology, Vol 31.

[76] Clauss A (1957). Gerinnungsphysiologische schnellmethode zur bestimmung des fibrinogens.. Acta Haematol.

[77] Hudson L, Hay FC (1976). Practical immunology.

[78] García-Ramallo E, Marques T, Prats N, Beleta J, Kunkel SL (2002). Resident cell chemokine expression serves as the major mechanism for leukocyte recruitment during local inflammation.. J Immunol.

[79] Aragón V, Díaz R, Moreno E, Moriyón I (1995). Characterization of *Brucella abortus* and *Brucella melitensis* native haptens as outer membrane O-type polysaccharides independent from the smooth lipopolysaccharide.. J Bacteriol.

